# Progress in Research and Application of Nanofiltration (NF) Technology for Brackish Water Treatment

**DOI:** 10.3390/membranes11090662

**Published:** 2021-08-28

**Authors:** Jiayu Tian, Xingrui Zhao, Shanshan Gao, Xiaoying Wang, Ruijun Zhang

**Affiliations:** 1 School of Civil and Transportation Engineering, Hebei University of Technology, Tianjin 300401, China; tjy800112@163.com (J.T.); zxr18632767623@163.com (X.Z.); gaoshanshan2018@126.com (S.G.); 2 School of Architectural Engineering, Sanming University, Sanming 365004, China; 20151099@fjsmu.edu

**Keywords:** brackish water, NF, desalination, treatment efficiency, combined process, membrane fouling, membrane cleaning

## Abstract

Brackish water is a potential fresh water resource with lower salt content than seawater. Desalination of brackish water is an important option to alleviate the prevalent water crisis around the world. As a membrane technology ranging between UF and RO, NF can achieve the partial desalination via size exclusion and charge exclusion. So, it has been widely concerned and applied in treatment of brackish water during the past several decades. Hereon, an overview of the progress in research on and application of NF technology for brackish water treatment is provided. On the basis of expounding the features of brackish water, the factors affecting NF efficiency, including the feed water characteristics, operating conditions and NF membrane properties, are analyzed. For the ubiquitous membrane fouling problem, three preventive fouling control strategies including feed water pretreatment, optimization of operating conditions and selection of anti-fouling membranes are summarized. In addition, membrane cleaning methods for restoring the fouled membrane are discussed. Furthermore, the combined utilization of NF with other membrane technologies is reviewed. Finally, future research prospects are proposed to deal with the current existing problems. Lessons gained from this review are expected to promote the sustainable development of brackish water treatment with NF technology.

## 1. Introduction

The shortage of freshwater has turned into one of the most critical concerns for global communities, especially for developing countries with a large population. It is estimated that the available freshwater only accounts for 0.8% of the total earth’s water [1]. At the same time, the deterioration of water quality accompanied by climate change and population growth further exacerbates this problem [2]. According to one estimation, 60% of the world’s population will live in water-deficient areas by the year 2025 [3].

In order to cope with the current water crisis, new sources of freshwater need to be constantly explored. During the past several decades, many water-deficient countries have been replenishing freshwater resources with desalinated water to meet the increasing demand [4]. Seawater accounts for more than 97.5% of the earth’s water resources, so the desalination of seawater with reverse osmosis (RO) has become the most popular choice of many coastal countries [5,6,7,8]. However, seawater reverse osmosis (SWRO) has some typical disadvantages, such as high operating costs and low water recovery, which is usually between 30 and 40% [9]. Besides, the highly concentrated brine brings potential hazard to the marine ecological environment [10]. To be different from seawater, brackish water is another important water resource with salinity ranging between fresh water and seawater [11,12]. As the salinity of brackish water is much lower than that of seawater, the osmosis pressure of brackish water is much lower than that of seawater, making brackish water desalination even easier and cheaper.

On the global scale, the evaporation rate of water is accelerating with global warming [13], so the salt concentration in the water of some local regions has increased. In addition, the melting of polar glaciers and rising sea levels cause the backflow of seawater into some coastal areas and islands [14]. In the low-lying areas with poor rock strata permeability and long groundwater runoff paths, once salts come into groundwater, the evaporation and concentration intensify the salt accumulation, thus converting fresh water into brackish water [15,16,17,18]. Furthermore, with the development of economy and industrialization, people discharge more and more solid waste and wastewater with high salinity into the environment. Once the salts in these wastes dissolve and concentrate in a specific water area, brackish water is also formed. All these factors are expanding the amount and distribution of brackish water. Taking China as example, brackish water is widely distributed in the arid areas of the northwest and the coastal areas of the southeast [19]. Globally, brackish water is also widely distributed in many countries, such as Saudi Arabia, Egypt, Turkey, United States and so on [1]. According to statistics, brackish water accounts for 1% of the total water on the earth, which is a huge potentially available water resource to be utilized [20].

Therefore, the desalination of brackish water has become an important approach to produce safe freshwater. Currently, RO and electrodialysis (ED) are the main technologies applied in brackish water desalination [21,22,23,24,25]. However, RO and ED have obvious shortcomings. For example, in the process of RO desalination of brackish water, almost all ions are removed. Although the harmful ions are rejected, some ions that are beneficial to body health are also removed [26]. Due to the high driving pressure in the RO desalination process, its energy consumption is high [27]. The long-term running of full-scale brackish water RO desalination plants reported by Ruiz-García et al. demonstrated that energy consumption is further increased due to RO membrane fouling [28]. Higher salt concentration increases the pressure required to produce water due to the osmotic pressure. Moreover, RO system water recovery is strictly limited by the composition of the inorganic salts in the feed water, because the scaling ions, such as Ca^2+^, Mg^2+^, SO_4_^2−^ and CO_3_^2−^, form inorganic precipitants (CaCO_3_, CaSO_4_, MgCO_3_, etc.) that can result in the RO membrane fouling and deteriorate the treatment efficiency [29,30]. At the same time, the brine produced by RO requires proper treatment [31]. When brackish water is desalinated by ED, energy consumption and desalination rate are closely related to the salt content of brackish water. High salt content obviously increases energy consumption and lowers the desalination rate. For instance, researching brackish water desalination by ED with a special carbon electrode, Xu et al. [32] found that the desalination rate decreased with the increase in brackish water salt concentration. Therefore, ED is more suitable for the desalination of brackish water with low salinity. In addition, because the organic matter and bacteria in raw water cannot be removed by ED, its application in the brackish water desalination project is limited.

Nanofiltration (NF) membranes are an effective, pressure-driven membrane separation process [33], which was developed in the late 1970s. The nanoscale pores in an NF membrane are usually less than 2 nm [34] and the molecular weight cut-off (MWCO) is typically between 150 and 800 Da [35]. NF is a separation process ranging between ultrafiltration (UF) and RO, which can partially remove the salt in water due to its size exclusion and charge exclusion [35,36,37], especially for sulfate and hardness ions [38]. Compared to RO, NF has numerous advantages, such as low operation pressure, low energy consumption and high throughput [39,40,41,42]. The comparison between RO and NF can be further referenced in a review published by Yang et al. [43]. In consideration of the features of NF technology, many researchers have carried out a series of studies on the desalination of brackish water with NF in recent years and explored the effects of different NF membranes and operating conditions, as well as those of operation modes on treatment efficiency. Up to now, numerous review papers focusing on the mechanism, application and development of NF technology have been published [44,45,46,47]. However, all these papers did not take the treatment of brackish water into consideration. Another review published in 2013 summarized the application and efficiency of NF membranes in the removal of ionic components from brackish water, sea water and brine disposal [48]. More recently, a review involving the improvements in membrane desalination processes for brackish water was published by Honarparvar et al. [49]. However, various membrane technologies, including RO, NF, ED and membrane capacitive deionization (MCDI), were all discussed and more attention was given on the separation mechanism. So, it is necessary to give an overview of the progress in research on and application of brackish water treatment with NF technology. To this end, this paper reviews the relevant practical cases in recent years, analyzes the factors affecting treatment efficiency, the membrane fouling problems and the corresponding control measures, as well as the combined utilization with other membrane technologies, and, finally, puts forward some suggestions for future research.

## 2. Brackish Water

**Figure 1:**
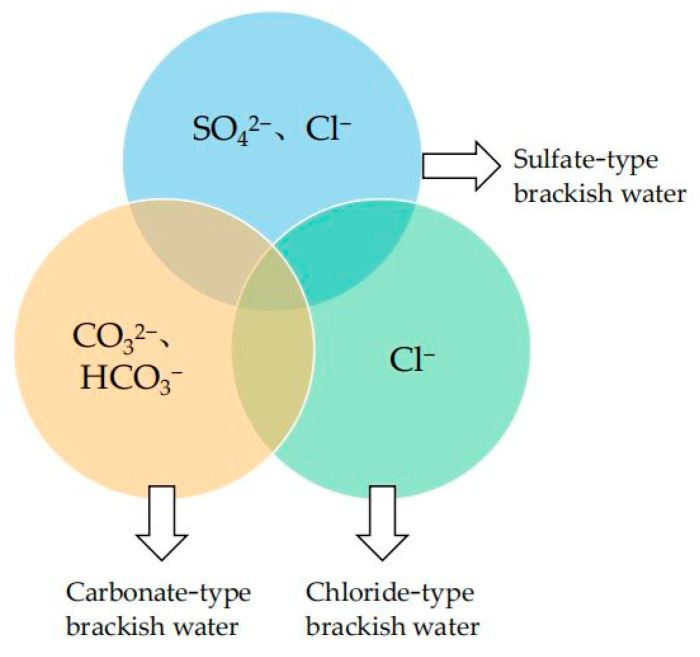
Chemical classification of brackish water. The common parts of the three-pie chart are K^+^, Na^+^, Ca^2+^ and Mg^2+^, etc.

Hydrogeology researchers commonly define brackish water as surface water or groundwater with the total dissolved solids (TDS) in the range of 1–25 g/L. The TDS of brackish water is higher than freshwater (TDS < 1 g/L) and lower than seawater (TDS ~35 g/L) [1,11]. Underground brackish water is relatively abundant, occupying more than half of the total groundwater storage [50]. Generally, brackish water with a TDS of 1–5 g/L is defined as low salinity brackish water, while brackish water with a TDS of 5–10 g/L is defined as medium salinity brackish water and brackish water with a TDS over 10 g/L is defined as high salinity brackish water. Brackish water tends to exhibit different chemical compositions depending on its geographical location and the composition of the stratum where it flows through. So, brackish water can also be classified into carbonate-type brackish water, sulfate-type brackish water and chloride-type brackish water according to its dominant ions, as shown in Figure 1.

Since brackish water is rich in salts such as MgSO_4_, CaSO_4_ and NaCl, it usually exhibits a bitter, salty and astringent taste. Among them, sulfates such as MgSO_4_ and NaSO_4_ play a major role in the bitterness of brackish water. Untreated brackish water is harmful to human health. Directly drinking untreated brackish water over a long period can cause some diseases such as digestive disorders, skin infections, dental fluorosis, hypertension, kidney stones and even various cancers [51,52]. Therefore, the quality standards for drinking water in different countries all stipulate concentration limits against many salt ions and TDS. Taking China as an example, 11 indexes (Arsenic, Cadmium, Chromium, Fluoride, Nitrate, Aluminum, Manganese, Chloride, Sulfate, TDS and total hardness) related to salt ions have been defined in the standards for drinking water quality, as shown in Table 1. Besides, agricultural irrigation with brackish water for a long time affects the permeability and water retention properties of the soil, thus inhibiting the growth of crops [53].

## 3. Factors Influencing the Efficiency of Brackish Water Treatment with NF

The efficiency of brackish water treatment with NF technology can be affected by various factors. These factors can be classified into three categories: characteristics of feed water, operating conditions and NF membrane properties. The detailed influence of these factors on the treatment efficiency is summarized and discussed as follows.

### 3.1. Characteristics of Feed Water

The efficiency of brackish water treatment with NF can be significantly affected by the characteristics of feed water, such as solution pH and ion composition, as well as concentration of organic matters. In the research by Song et al. [54], model brackish water was treated by a commercial NF membrane in which the pH, TDS and TOC of the feed water were regulated by citric acid, NaOH, inorganic salt and humic acid (HA), respectively. It was found that, when pH > 6, the removal rate of inorganic salts improved with the increase in pH. When the TDS was gradually increased from 1 to 4.5 times, the specific permeate flux decreased by 9.1% and the removal of TDS decreased by 18.21%. With the increase in TDS, the decreased permeate flux and TDS removal was resulted by the enhanced concentration polarization and decreased effective trans-membrane pressure. When the TOC concentration increased from 1 to 4.5 times, the specific permeate flux declined by 8.78%. Moreover, an interesting phenomenon observed was that the removal of Mg^2+^, Ca^2+^ and TDS was increased by 74.13%, 48.14% and 20.50%, respectively. Similarly, Su et al. [55] studied the influence of DOC in feed water on the treatment of model brackish water by NF. The water quality of the model brackish water was similar to the brackish groundwater in the Huanghuai region of China. In their research, the DOC was regulated by changing the HA concentration. It was found that, when the feed DOC increased from 0.54 mg/L to 2.43 mg/L, the specific permeate flux declined by 5.93%, while the growth in the removal rate of Ca^2+^, Mg^2+^, SO_4_^2−^ and CO_3_^2−^ was 47.23%, 58.54%, 98.75% and 82.59%, respectively. It can be explained by the complexation interaction between HA and Ca^2+^ [56,57,58]. On one hand, Ca^2+^ can partially neutralize the negative charge of HA molecules [59]. On the other hand, the complexation bridging between HA and Ca^2+^ can promote the formation aggregates with larger molecular weights [60]. Consequently, more organic foulants deposit on the membrane surface and form a more compact cake-layer, which can produce resistance to the mass transfer of both water and ions [61,62]. With the increase in HA concentration, the cake layer on the membrane surface was gradually thickened, resulting in a decrease in specific permeate flux and an increase in ion rejection.

According to the above-discussed cases, the influence of feed water characteristics on treatment efficiency can be concluded: (1) High solution pH can improve the desalination rate because of the enhanced charge exclusion between the NF membrane and negatively charged ions. However, it should be noted that a higher solution pH promotes the precipitation of scaling ions, such as CaCO_3_. Besides, strong alkaline condition may damage the cross-linking structure of NF membranes [63]. (2) High TDS concentration in feed water reduces the effective trans-membrane pressure and enhances the ion concentration polarization, which can obviously decrease the permeate flux and ion rejection. Meanwhile, the operating pressure would need to be increased to overcome the osmosis pressure induced by high TDS, which would increase energy consumption and running costs. In addition, the scaling ions in TDS would increase the formation of inorganic fouling, such as the precipitation of CaSO_4_. Therefore, proper pretreatment may be necessary to remove these scaling ions and reduce the feed TDS. The influence of pH and salt on the NF separation process can be further referenced in the review provided by Luo et al. [64]. (3) Although many studies found that the organic matters in feed water can obviously increase the ion rejection rate, it should be noted that this enhancement may not be long-term effective. The membrane fouling induced by organic matters cannot only increase the mass transfer resistance and decrease the water flux, but also reduce the salt rejection via the cake enhanced concentration polarization (CECP) [65,66]. Therefore, for long-term stable operation, the influent TOC concentration should not be too high. Pretreatments aiming for the removing the organic matters in feed water should be taken into consideration.

### 3.2. Operating Conditions

In addition to the characteristics of feed water, the treatment efficiency can also be changed by regulating the operating conditions, such as operating pressure, cross-flow velocity and operating temperature. For instance, Zhang et al. [67] studied the influence of operating pressure on the treatment of model brackish water with an NF membrane in which brackish water consisted of 10 g/L NaCl. It was found that the permeate flux was increased by 58%, when the operating pressure increased from 1.2 MPa to 1.4 MPa. Meanwhile, the desalination rate was improved from 95% to 97%, when the operating pressure increased from 1.1 MPa to 1.5 MPa. As the operating pressure increased, the net driving force of the desalination process increased and so did the permeate flux. However, according to the solution-diffusion model, the salt permeation process is proportional to the concentration difference across the membrane, independent of the operating pressure. Therefore, with the increase in operating pressure, the increase in water permeation dilutes the ion concentration of the permeate solution and increases the observed salt rejection. Similarly, Song et al. [68] tested the treatment efficiency of two commercial NF membranes under different operating conditions. The results showed that the permeate flux and the removal rate of divalent ions (Ca^2+^, Mg^2+^), as well as TDS, were improved with the increase in operating pressure or cross-flow velocity. At the same time, with the increase in feedwater temperature (7–35 °C), the permeation flux increased, while the rejection rates of Ca^2+^ and Mg^2+^ decreased greatly. The increase in temperature reduced the viscosity coefficient of the solution, resulting in the increase in water diffusivity. Therefore, based on the solution-diffusion model, the permeate flux increased. There are two reasons for the decreased removal rate of Ca^2+^ and Mg^2+^. On one hand, with the increase in feedwater temperature, the decrease in pore density and the increase in pore size are expressed as pore enlargement [69,70]. On the other hand, these structural changes affect the solute permeation and activation energies of permeation, in which solute permeation is proportional to pore density [71].

In general, the influence of operating conditions on treatment efficiency can be obtained as follows: (1) Although higher operating pressure can improve permeate flux and treatment efficiency, it should be noted that membrane fouling would be aggravated once the permeate flux exceeds a critical value [72]; so, the pressure should not be too high. (2) Higher cross-flow velocity can reduce the thickness of the boundary layer and mitigate the concentration polarization, thus reducing membrane fouling [73]. However, excessive cross-flow velocity would increase energy consumption and may also cause damage to the selective layer. So, for long-term stable operation, cross-flow velocity should be controlled at an optimized value. (3) When the feed water temperature is too low, the permeate flux is small. However, when the temperature is too high, the ion removal rate would be decreased. Normally, the temperature of feed water should be controlled between 15 and 30 °C.

### 3.3. Properties of NF Membrane

As the core of NF technology, the properties of the NF membrane itself can certainly influence the efficiency of brackish water treatment. In detail, the pore size and surface charge of a specific NF membrane are expected to yield different permeate flux and salt rejection. In previous publications, the famous NF90 and NF270 produced by DOW were usually employed to study the differences in treatment efficiency. The main characteristics of NF90 and NF270 are shown in Table 2. The surface of most commercial NF membranes is negatively charged [74]. So, the removal rate of anion is generally higher because of the electrostatic repulsion between the negatively charged ion and membrane matrix [68]. The ion rejection rate and permeate flux are also influenced by the membrane pore size. In general, a low rejection rate is associated with high water permeability, which can be described as the “trade-off” between water permeability and desalination rate [46].

In the study by Hilal et al. [76], NF90 and NF270 were adopted to treat the model brackish water prepared with NaCl. The results showed that the permeate flux of NF90 was lower than that of NF270. Conversely, the rejection rates of NF90 and NF270 to the 5000 ppm NaCl solution were 95% and 29%, respectively, when the operating pressure was fixed at 0.9 MPa. The above phenomenon can be explained by the steric hindrance mechanism, because the pore size of NF90 was smaller than that of NF270, so the permeate flux was lower, while the rejection rate was higher. Similarly, the removal rate of NF90 for Na^+^, Mg^2+^ and Ca^2+^ was significantly higher than that of NF270 [2]. Ramdani et al. [77] studied the defluorination performance of NF90 and NF270 for the treatment of Algerian brackish water. They found a similar phenomenon where the permeate flux of NF90 was lower than that of NF270. However, the difference in removal rates of fluoride ions was not big, which were 88% and 79% for NF90 and NF270, respectively. Moreover, the fluoride concentrations of the permeate water were 0.35 mg/L and 0.62 mg/L, respectively. These two values were both lower than the Algerian standards. Under the circumstances, NF270, which showed a higher permeate flux and lower TDS removal rate, may be the optimal selection, because it is expected to yield lower energy consumption and slighter membrane fouling on the premise of meeting the fluoride removal requirement. This should also be the principle for the selection of membrane type when treating other brackish water with NF technology.

Direct design and fabrication of a novel NF membrane is a significant route for promoting the water treatment efficiency. In fact, substantial efforts have been made in developing novel and high-performance NF membranes, which can be referenced in the reviews on this topic [78,79]. However, few relevant reports can be found aiming for the treatment of brackish water as far as we know. So more research about this issue is necessary in the future.

## 4. Membrane Fouling Control during the Treatment of Brackish Water with NF

Membrane fouling is an inevitable problem faced by all membrane applications, including the treatment of brackish water with NF. In order to mitigate membrane fouling and keep long-term stable operation, various measures can be taken according to actual conditions. Membrane fouling refers to the phenomenon that the adsorption and deposition of rejected foulants (including particles, colloid, inorganic salts, organic matters and microorganisms) on the membrane surface or in the membrane pores, which can decrease the permeate flux, change the effluent quality and increase energy consumption and operating costs [80,81,82]. In general, NF membrane fouling during the treatment of brackish water can be reduced by three aspects, including pretreatment and optimization of operating conditions, as well as the preparation or selection of anti-fouling membrane materials.

### 4.1. Pretreatment

Membrane fouling is largely affected by the feed water characteristics. In order to enhance the brackish desalination performance of an NF membrane, feed water should be pretreated with various technologies to remove some typical foulants; then, membrane fouling could be mitigated. At present, the commonly used pretreatments for the treatment of brackish water with NF can be classified into three categories, including conventional water treatment process, membrane filtration and the adjustment of solution chemistry.

**Figure 2:**
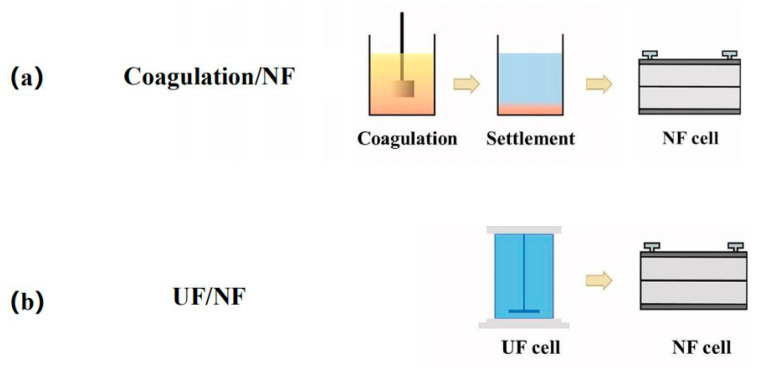
Schematic diagram of pretreatment process for NF: (**a**) coagulation/NF process; (**b**) UF/NF process. Reproduced with permission from Reference [86]. Copyright 2020, Elsevier.

Conventional water treatment processes, including coagulation, adsorption and sand filtration, can all be taken as the pretreatments of brackish water desalination with NF, because these processes can remove various components to different extent. Among these processes, coagulation is the most widely used one [83,84], in which the colloidal matters and hydrophobic foulants can be effectively removed via the formation and removal of floc, just as shown in Figure 2a. This method is simple and economical, but it should be noted that the residual coagulant may inversely aggravate membrane fouling, when the coagulant dosage is not properly controlled.

Membrane filtration, including MF and UF, is also a good option for pretreatment. Unlike coagulation, membrane filtration does not require the addition of any chemicals to remove contaminants. Among them, UF is the most widely used one [85]. As shown in Figure 2b, UF can reduce membrane fouling by removing the suspended solids, colloids, bacteria and viruses and even organic matters with high molecular weight, such as proteins and natural organic matters (NOMs). The high efficiency and stable performance of UF make it rather competitive, but the fouling of the UF membrane itself should also be taken into consideration.

Solution chemistry can also obviously influence membrane fouling. So, the regulation of solution chemistry is another important pretreatment option. For instance, Ca^2+^ and Mg^2+^ with bridging effects with humic acid and sodium alginate can obviously aggravate membrane fouling [87,88], so membrane fouling is expected to be mitigated by removing Ca^2+^/Mg^2+^ or humic acid/sodium alginate. In addition, the solution chemistry can also be regulated by adjusting the pH. It was found that the charge density of humic acid increased with the increase in pH [62]. Moreover, with increasing pH, the streaming potential of the membrane changes from positive to negative, resulting in an increase in electronegativity [89,90]. So, at a high solution pH, the electrostatic repulsion between humic acid molecules or between humic acid and membrane can be enhanced, resulting in the mitigation of membrane fouling. Sodium alginate also shows a similar phenomenon [91]. At the same time, BSA is also susceptible to the solution pH and membrane fouling induced by BSA was found to be most serious at its isoelectric point (IEP_BSA_ = pH 4.7) [92,93]. Therefore, the solution pH should be adjusted to a weak alkaline to prevent the occurrence of BSA fouling. However, it should be noted that inorganic fouling is also influenced by the solution pH; a high solution pH aggravates the formation of inorganic precipitants (such as CaCO_3_ and Mg (OH)_2_) [94,95]. So, the regulation of the solution chemistry should be cautiously determined according to the quality features of raw water.

### 4.2. Optimization of Operating Conditions

Membrane fouling is also closely related to the operating conditions, such as permeate flux and cross-flow velocity. Numerous studies have shown that membrane fouling can be aggravated at high pressure and low cross-flow velocity. An important concept related to the operating condition is the critical flux. In the filtration process, if the permeating flux does not exceed the critical flux, membrane fouling can be controlled in the rather low extent. Once the permeating flux exceeds the critical flux, the fouling process is greatly accelerated [96,97,98,99]. Therefore, it is desirable to operate the membrane system below the critical flux to avoid serious membrane fouling. Moreover, the concentration polarization increases when cross-flow velocity decreases, because the shear force on the membrane surface decreases with the decrease in cross-flow velocity [100]. So, improving cross-flow velocity is an effective strategy for mitigating the membrane fouling, although it can increase energy consumption.

System water recovery is another critical parameter to be optimized. Higher system recovery can improve the permeate water yield, while, on the other hand, it can aggravate the concentration polarization and promote the formation of scaling (inorganic membrane fouling) on the membrane surface. Calculation predicting is meaningful to the determination of the water recovery. For instance, a study reported by Mitko et al. assessed the scaling risk in the treatment of mine brackish water, in which a method for estimating the maximum water recovery before the occurrence of the calcium sulfate dihydrate scaling was proposed [101].

### 4.3. Selection of NF Membranes with Anti-Fouling Ability

Membrane fouling is essentially a kind of physical and chemical phenomenon on the NF membrane surface. Many researchers have speculated that the properties of the membrane surface could determine the interaction between the membrane and foulants [102]. Therefore, membrane fouling can be effectively alleviated by regulating the features of the membrane surface [103]. For NF membranes, some studies have found that enhancing hydrophilicity or reducing surface charge density and roughness can reduce the accumulation of foulants; then, the membrane anti-fouling ability can be enhanced [104,105].

In recent years, with the rapid development of material science, a variety of novel materials have been used to prepare anti-fouling NF membranes. For instance, in the study by Wu et al. [106], ZIF-8 and GO were employed to synthesize the rGO/ZIF-8 composite nano-materials by hydrothermal method; then, the self-made composite nano-materials (rGO/ZIF-8) were introduced into the functional layer of an NF membrane by interface polymerization (IP). The separation and anti-fouling performance of the modified membrane for treatment of actual brackish water was analyzed. It was found that when the amount of rGO/ZIF-8 was 0.005 wt%, the hydrophilicity of the modified membrane reached the highest level and electronegativity also increased. The organic foulants (BSA, SA and HA) were not easy to accumulate on the membrane surface. Correspondingly, after rinsing with deionized water, the flux recovery of the fouled modified membrane was more than 80%, which was much higher than that of the unmodified membrane.

**Figure 3:**
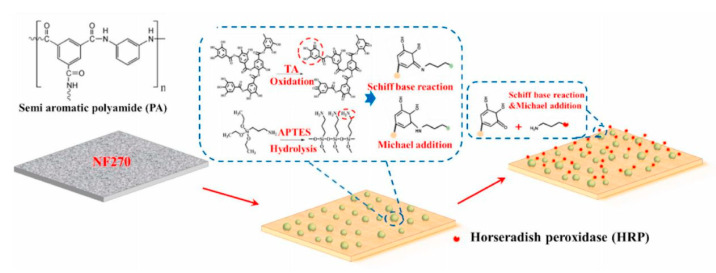
Schematic illustration of TA-APTES coating and HRP immobilization on an NF membrane. Reproduced with permission from Reference [110]. Copyright 2021, Elsevier.

Surface modification is another effective strategy for enhancing the membrane antifouling performance [107,108]. For example, in the study by Seman et al. [109], they selected neutral hydrophilic N-vinylpyrrolidone as the monomer and modified the surface of a commercial NF membrane by grafting the monomer onto the surface using the UV-photografting polymerization technique to reduce the fouling trend. In the study by Zhang et al. [110], a commercially available NF membrane was modified by tannic acid-3-aminopropyltriethoxysilane (TA-APTES) coating and subsequent horseradish peroxidase (HRP) immobilization, as shown in Figure 3, which improved the antifouling performance of the NF membrane. However, no relevant research aiming for the treatment of brackish water can be found up to now. Allowing the commonality of membrane surface modification, surface modification would be promising in enhancing the antifouling ability of NF membranes fitting for the purpose of brackish water treatment. More research about this issue is necessary for future study.

## 5. Membrane Cleaning

Membrane fouling can be mitigated rather than being absolutely avoided. Once the membrane fouling reaches a certain extent, membrane cleaning is necessary. Generally, the water yield, effluent quality and the pressure drop degree of the membrane can be used to determine whether the membrane needs to be cleaned. Judgment is mainly based on the following parameters: (1) under normal feed water pressure, water production decreases by 10–15%, compared with the normal value, (2) the water quality of produced water decreases by 10–15% or the salt permeability increases by 10–15% and (3) in order to maintain normal water production, the operating pressure after temperature correction is increased by 10–15%.

Membrane cleaning efficiency can be evaluated by membrane permeate flux recovery (FR) or resistance removal rate (RR), as shown in Equations (1) and (2) [111,112]. Meanwhile, AFM, SEM, hydrophilicity and FTIR spectroscopy of the NF membrane before and after cleaning can be used together with FR and RR to more comprehensively evaluate the cleaning performance.

*FR* (%) = (*j_pc_*/*j_pi_*) × 100 (1)

*RR* (%) = [(*R_f_ − R_c_*)/(*R_f_* − *R_i_*)] × 100 (2)

where *j_p__i_* is the initial permeate flux value of the new compacted membrane, *j_pc_* is the permeate flux after membrane cleaning and *R_i_*, *R_f_* and *R_c_* are the initial resistance, the resistance after fouling and the resistance after membrane cleaning, respectively.

Membrane cleaning includes physical cleaning, chemical cleaning and enzymatic cleaning. Among them, for the NF membrane fouled by brackish water, the commonly used cleaning methods are physical cleaning and chemical cleaning.

### 5.1. Physical Cleaning

**Figure 4:**
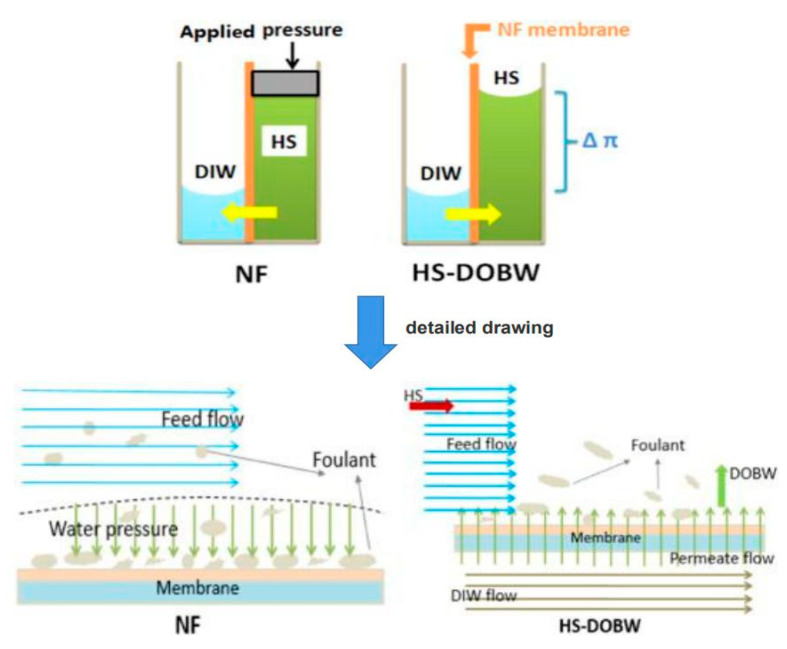
Schematic of NF membrane fouling and HS-DOBW cleaning. Reproduced with permission from Reference [115]. Copyright 2015, Elsevier.

Physical cleaning methods include the hydraulic method, gas-liquid pulse, backwashing, circulating washing and osmotic backwashing. Among them, the hydraulic method and osmotic backwashing are the most widely used ones in NF desalination of brackish water [113,114]. The hydraulic method is to remove foulants attached on the membrane surface by reducing the operating pressure and flushing the membrane surface with an aqueous solution for a certain time. This method is the simplest, but the membrane permeate flux recovery is relatively low and the membrane flux drops rapidly again after short-term operation. As shown in Figure 4, osmotic backwashing is achieved by adding a highly concentrated salt solution (HS) to the feed side of the membrane and adding deionized water (DIW) to the permeate side. A large osmotic pressure driving force is formed across the membrane to make the water flow from the permeate side to the feed side. When water passes through the membrane, the driving force promotes the removal of the foulants on the membrane surface. This is an environmentally friendly cleaning method with high cleaning efficiency.

### 5.2. Chemical Cleaning

**Figure 5:**
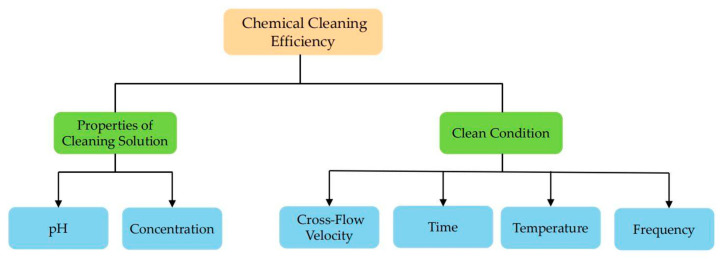
The classification of factors affecting chemical cleaning efficiency.

In long-term operation, once physical cleaning cannot restore the membrane flux to the initial value, a chemical cleaning method is used. Chemical cleaning uses chemical reagents to react with substances that cause membrane fouling to restore membrane flux. Typically, the agents used for chemical cleaning include sodium hydroxide (NaOH), sodium dodecyl sulfate (SDS), sodium ethylenediaminetetraacetic acid (EDTA) and sodium hypochlorite (NaClO). The factors affecting chemical cleaning efficiency are shown in Figure 5.

**Figure 6:**
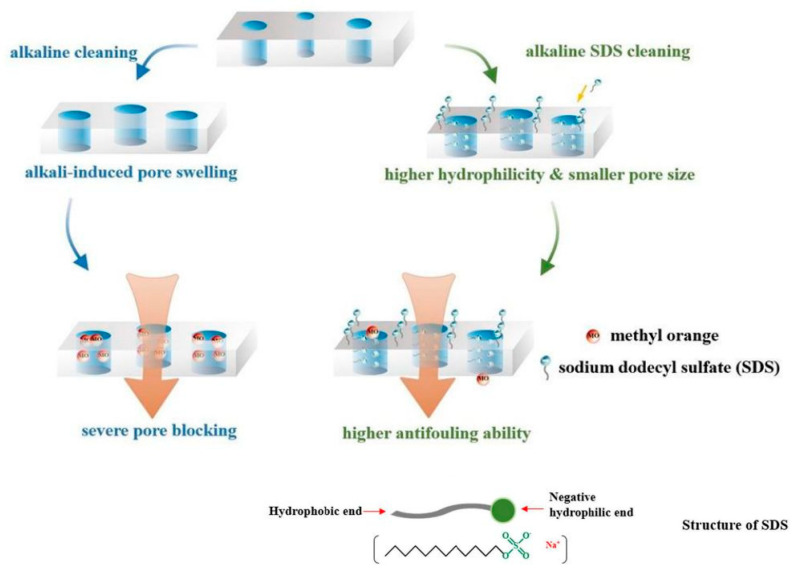
Illustration of alkaline cleaning and alkaline SDS cleaning for the methyl orange induced membrane fouling. Reproduced with permission from Reference [122]. Copyright 2021, Elsevier.

Chemical cleaning has a high cleaning efficiency for organic fouling and biofouling [116,117]. Many studies have shown that organic fouling is the first step in NF membrane fouling [118] and it plays an important role in promoting other membrane fouling (inorganic fouling, colloid fouling and biofouling) [119,120,121]. The cleaning of organic fouling commonly requires the joint application of lye and surfactant and the cleaning mechanism is as follows: Due to the high pH and ionic strength of NaOH in the alkali solution, the counterion concentration around the membrane matrix increases and forms a counterion layer, which causes charge repulsion, then the membrane pores expand. At the same time, the surfactant-sodium dodecyl sulfate can reduce the interfacial tension of lye and improve its wetting permeability, so that lye can quickly penetrate into the interior of the foulant layer. Finally, organic matter is saponified into soluble substances, which can be easily removed. It is worth noting that alkaline washing alone enlarges the membrane pores, then small solutes are more prone to enter the pores, resulting in more serious pore narrowing effect and membrane fouling, while adding SDS to the alkaline cleaning agents, as shown in Figure 6, SDS not only enhances the cleaning efficiency via its strong binding ability, but also offsets the alkali-induced pore swelling to further improve the antifouling performance of the NF membrane [122].

Due to the complex and diverse composition of membrane fouling, a single cleaning method is usually unable to achieve the desirable cleaning efficiency. Therefore, in practical engineering, different cleaning methods are often used in combination to better restore the performance of NF membranes. Typical examples related to the treatment of brackish water are shown in Table 3. More information can be found in the review focusing on the topic of NF membrane cleaning [123].

## 6. Combined Utilization of NF with Other Membrane Technology

In the previous section, the effects of feed water characteristics, operating conditions and membrane properties on the treatment efficiency of brackish water are discussed. In fact, NF technology can also be combined with other membrane technologies to form different combined membrane process systems, so as to improve permeate water quality and water recovery, as well as reducing energy consumption and membrane fouling. Examples of practical applications are presented below to illustrate the feasibility of the combined process for desalination of brackish water.

### 6.1. Combined Application of NF and UF

**Figure 7:**
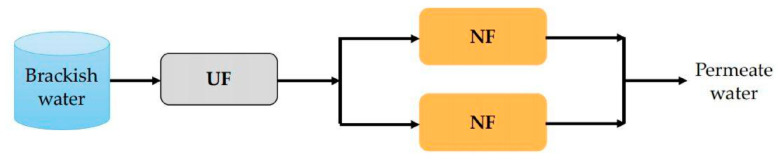
Schematic diagram of the UF–NF system.

In recent years, low-pressure membrane processes such as ultrafiltration (UF) and microfiltration (MF) have been used as pretreatments of NF technology in the treatment of brackish water. In particular, UF membranes are receiving increasing attention due to their excellent removal efficiency of colloidal particles, macromolecular organic matters, bacteria and viruses, which can induce membrane fouling [125]. For instance, in the study by Fan et al. [85], the UF/NF dual-membrane system, as illustrated in Figure 7, was applied to treat high chlorine brackish water taken from the Mount Yan. It was found that the dual-membrane system had a good removal efficiency on organics and salts and the removal rate of chloride was up to 95%. Although the fouling mitigation effect of the UF pretreatment was not mentioned in this research, it can be anticipated that the UF membrane pretreatment could reduce the fouling tendency which has been confirmed by many other studies. For example, in the study by Song et al. [126], UF modules were taken as the pretreatment of seawater. It was found that the NF membrane fouling was obviously mitigated because the SDI_15_ and turbidity of the raw seawater were effectively decreased after UF pretreatment.

### 6.2. Combined Application of NF and NF

**Figure 8:**
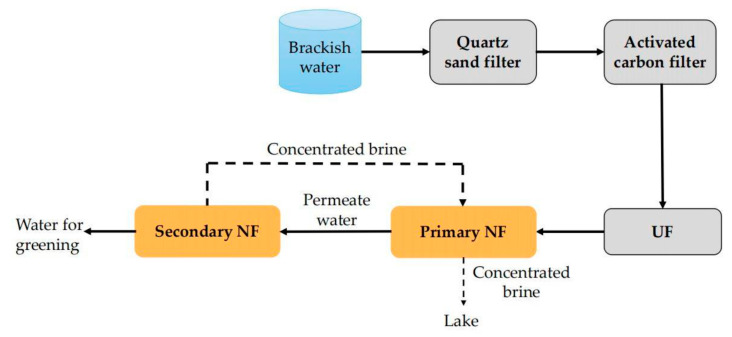
Flow chart of two-stage NF process.

For brackish water with high salinity, the desalination rate of single-stage NF usually cannot meet the desalination requirements, while the energy consumption of RO treatment is high [127,128]. So, the researchers set their sights on the dual-stage NF which indeed is the combined application of NF and NF. For example, Chen et al. [129] designed a dual-stage NF system, as illustrated in Figure 8, to desalinate surface brackish water in Binhai New Area of Tianjin (TDS > 12,000 mg/L). In this system, the quartz sand filter, activated carbon filter and UF acted as the pretreatment units. The brackish water after pretreatment came to the NF–NF unit for desalination. The results show that the salt content, total hardness and COD of the permeate water of this system were 600 mg/L, 10 mg/L and 1.5 mg/L, respectively. These water quality parameters can meet the “Water quality standard for non-potable urban use” (GB/T 18920-2002) proposed by China. More importantly, this dual-stage NF system yields better permeate water quality than the single-stage NF process and lower energy consumption than the RO process. So, it has a good demonstration and guidance role in this region and similar cases of brackish water desalination.

### 6.3. Combined Application of NF and RO

**Figure 9:**
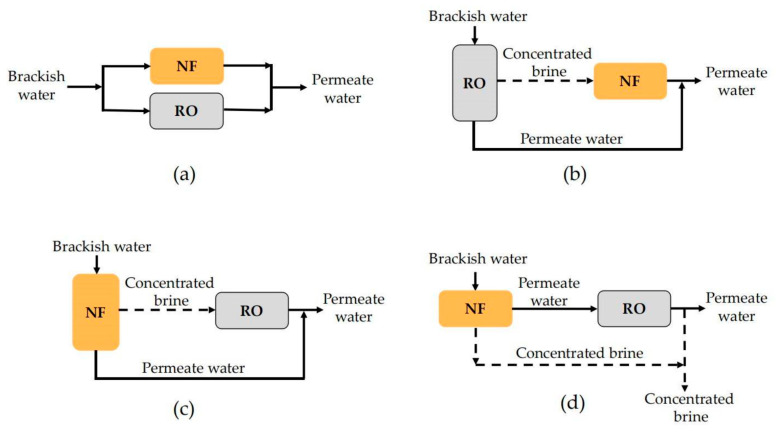
Schematic diagram of four hybrid systems configuration: (**a**) parallel NF/RO system; (**b**) RO-C-NF system; (**c**) NF-C-RO system; (**d**) tandem NF–RO system.

NF technology can be used together with RO to maximize their respective advantages and achieve the optimal effect of brackish water treatment. In this way, permeate water quality or system water recovery can be improved. Up to now, four different forms of NF and RO combinations have been proposed, which can be denoted as the parallel NF/RO system, the RO-concentrate-NF system (RO-C-NF), the NF-concentrate-RO system (NF-C-RO) and the tandem NF–RO system, respectively. The flow diagram of each combination process is shown in Figure 9.

In the parallel NF/RO system illustrated in Figure 9a, brackish water enters the NF and RO membrane systems at the same time and the filtrate of each membrane system is collected together to form mixed permeate water. In the RO-C-NF system shown in Figure 9b, the brackish water first enters the RO system; then, the concentrated brine of RO is treated by the NF membrane. Finally, the permeate water of RO and NF are mixed to form the permeate water. In the NF-C-RO system shown in Figure 9c, the brackish water first enters the NF system; then, the concentrated brine of NF is treated by RO. The permeate water of NF and RO are mixed to form the final permeate water. Cai et al. constructed a pilot-scale NF-C-RO system to desalinate natural ground brackish water containing high Sr concentration (10.3 mg/L) in Tanzania [130]. It was found that the NF unit only had a Sr rejection of 55–67%, while RO can remove almost 100% of Sr, so the mixture of NF permeate and RO permeate can meet the USEPA guideline of 1.5 mg/L. In the research by Srivastava et al. [131], TDS removal and water recovery of the three combinations described in Figure 9a–c were compared. It was found that the water recovery of the parallel NF/RO system was the highest. The water recovery of the RO-C-NF system was higher than that of the NF-C-RO system. The TDS removal rate of the RO-C-NF system was the lowest among the three combined systems, while the NF-C-RO system presented the highest TDS rejection.

Another combination form, denoted as tandem NF–RO system, is illustrated in Figure 9d. In this process, NF technology is indeed taken as the pretreatment of brackish water desalination with RO, which is expected to prolong the running life of the subsequent RO by reducing the scaling tendency. A retrofitting feasibility assessment made by Ruiz-García et al. confirmed that the utilization of NF before RO could effectively promote the efficiency, permeate quality and economic viability of the plant [132]. Talaeipour et al. [33] tried three membrane processes, including single NF, single RO and tandem NF–RO system, to treat brackish water from Qom Province in Iran. After treatment with single NF, single RO and tandem NF–RO system, the system desalination rates were 50.21%, 72.02% and 78.65%, respectively; the removal rates of Cl^−^ were 21.10%, 43.8% and 63.95%, respectively.

### 6.4. Combined Application of NF and FO

**Figure 10:**
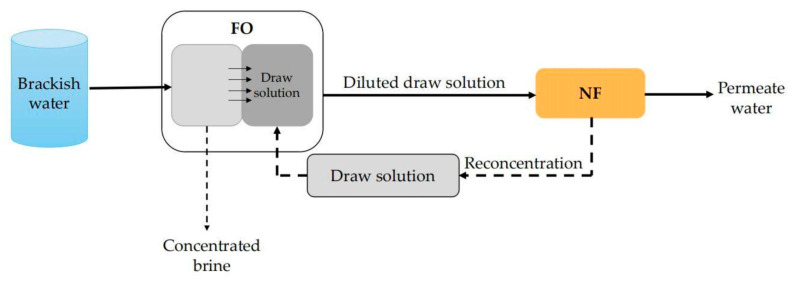
Schematic diagram of FO–NF system.

To be different from many other pressure-driven membrane separation processes, FO is driven by the osmotic pressure difference across the membrane [133]. High water recovery rate, high desalination rate and low membrane fouling tendency are typical advantages of FO technology [134,135,136]. For this reason, more and more scholars have applied FO to the desalination of brackish water. For example, Zhao et al. [137] applied the FO–NF system, as shown in Figure 10, to desalinate brackish water (TDS, 3970 mg/L) from Lake Mawson, South Australia. In this system, a Na_2_SO_4_ solution was used as the draw solution. The working process is described as follows: Brackish water first entered the FO system in which the water molecules came across the FO membrane driven by the osmosis pressure generated by the high concentration of the Na_2_SO_4_ solution. Meanwhile, the Na_2_SO_4_ solution was diluted by the permeate water of FO. Then, the diluted Na_2_SO_4_ solution came to the NF system for further desalination. As the Na_2_SO_4_ rejection of the selected NF membrane (NF270) was rather high, the TDS of permeate water of the NF process was very low. Moreover, the concentrated water (high concentration of Na_2_SO_4_) of the NF process can be recycled to the FO system as the drawn solution. It was found that the removal rate of TDS of this FO–NF system was up to 97%. However, when the same brackish water was directly desalinated with NF270, the TDS removal rate was only 35.5%. In addition to the higher TDS removal, FO membrane can also effectively remove other foulants, such as colloidal particles, organic matters and scaling ions. Theoretically, only Na_2_SO_4_ can appear in the feed water of an NF membrane. So, membrane fouling of the NF unit is expected to be greatly reduced.

### 6.5. Other Combinations

**Figure 11:**
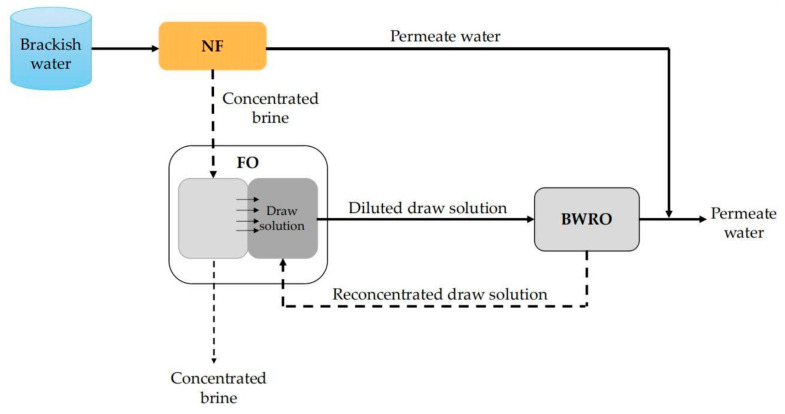
Schematic diagram of NF–FO–BWRO system.

NF technology can also be combined with multiple membrane processes to achieve efficient treatment of brackish water. For example, Altaee et al. [138] proposed a NF–FO–BWRO desalination system to improve the recovery rate of the system (Figure 11). In this system, brackish water firstly entered the NF unit to remove most of divalent ions and produced the first stage permeate with a relatively low energy consumption. In consideration of the relatively high TDS of the permeate water of this stage, it was mixed with the BWRO permeate water with much lower TDS to form the final permeate water. The concentrated brine of the NF unit was treated by the FO system with NaCl as the draw solution. So, the water in the NF brine could be extracted into the NaCl draw solution, which was then desalinated by the RO unit. The results show that the recovery rate of the NF–FO–BWRO system for brackish water with salt content of 1–2.4 g/L was up to 90%, which was much higher than the conventional single BWRO system, with a recovery rate around 70%. The high recovery rate of the NF–FO–BWRO system can be attributed to the employment of FO, which can simultaneously concentrate the NF brine and utilize the RO brine.

## 7. Conclusions and Future Perspectives

This paper presents a comprehensive review of the research progress and applications of NF technology for brackish water treatment in recent years. The application development and advantages of NF membranes are introduced. Then, the definition, classification and formation of brackish water are expounded, followed by an overall discussion on the desalination of brackish water with NF technology. The discussion focuses on the factors affecting the efficiency of brackish water treatment by NF and the membrane fouling that occurs in the process of brackish water treatment with NF is also briefly discussed. A series of fouling control strategies and cleaning methods are analyzed. Finally, the combined application of NF with other membrane technologies are reviewed. In order to promote the sustainable development of brackish water treatment with NF technology, more attention should be focused on the following in future research:

(1) The characteristics of specific brackish water and the difference in purification objectives should be taken into account first, when selecting the treatment process. Moreover, a comprehensive comparison is needed from the perspectives of economy, technology and environment;
(2) Understanding the membrane fouling mechanism is the foundation of membrane fouling control, but most of the reported research related to the desalination of brackish water with NF only concerns the treatment efficiency; the analysis of the fouling mechanism is not sufficient. So, more microscopic membrane characterizations are necessary to be adopted to promote the deep understanding of membrane fouling;
(3) As the core of NF membrane technology, membrane material should be given more attention to further improve the permeability, selectivity and stability, as well as reducing manufacture costs. In addition, membrane fabrication should be “fitting for purpose” according to the specific brackish water quality and treatment requirements;
(4) The application of NF technology integrated with other emerging water treatment technologies is expected to further improve the performance of brackish water treatment. For instance, an integrated NF-calcite contactor process proposed by Haddad et al. was proved to be a feasible method to effectively remove the undesirable compounds (particularly manganese (Mn), iron (Fe) and hardness) from groundwater [139]. Therefore, more novel hybrid brackish water treatment process combination with NF as core should be explored.
(5) In the process of treating brackish water, a large amount of concentrated brine is produced. Up to now, most of the concentrated brine has been directly discharged into the environment, which brings a great potential harm to ecology. So, subsequent treatment of concentrated brine is an important issue to be focused on in future research. The high concentration of salt in the brine should be regarded as a resource, rather than a contaminant.

## Figures and Tables

**Table 1:** Indicators for drinking water quality established by China.

Indicators	Values
Arsenic (mg/L)	0.01
Cadmium (mg/L)	0.005
Chromium (hexavalent, mg/L)	0.05
Fluoride (mg/L)	1.0
Nitrate (in N, mg/L)	10
Aluminum (mg/L)	0.2
Manganese (mg/L)	0.1
Chloride (mg/L)	250
Sulfate (mg/L)	250
TDS (mg/L)	1000
Total hardness (in CaCO_3_, mg/L)	450

**Table 2:** Characteristics of NF 90 and NF270 [75].

Items	NF90	NF270
Surface layer material	fully aromatic polyamide	semi-aromatic polyamide
Water permeability (L/(m^2^·h·bar)) ^a^	9.0	17.5
Salt rejection (%) ^b^	87.4	56.3
Zeta potential (mV) ^c^	−13	−53
Contact angle (°) ^d^	55.2 ± 2.5	18.3 ± 2.6
Surface roughness (nm)	64.9 ± 8.1	5.1 ± 0.5
Pore radius (nm) ^e^	0.31	0.40

^a^ Water permeability was recorded after 24 h membrane compaction under the following conditions: 100 psi, 25 °C, 1 L/min, pH of 7.5 and DI water. ^b^ Salt rejection was determined after NaCl was added to the feed tank for 24 h under the following conditions: 100 psi, 25 °C, 1 L/min, pH of 7.5 and 10 mM NaCl. ^c^ Zeta potential was measured at pH = 7.5 with 10 mM NaCl as the background electrolyte. ^d^ The contact angle and surface roughness were measured for the vacuum-dried membrane coupons after 24 h exposure in DI water. ^e^ The membrane effective pore radius was calculated according to a solute transport model.

**Table 3:** Typical membrane cleaning examples related to the treatment of brackish water.

NF Membrane	Feedwater	Cleaning Method	Results	Reference
DK	Synthetic brackish water	2.0 wt.% citric acid + NaOH solution (pH = 10)	FR = 97.80%	[55]
			RR = 61.63%	
DL			FR = 95.40%	
			RR = 49.35%	
NF3A	Synthetic arsenic-rich brackish water	Citric acid (pH = 3) + ultrasound (with the power intensity of 1 W/cm^2^)	FR = 99.99%	[124]
NF-1812	Synthetic brackish water	Hydraulic cleaning + 0.1% NaOH + 0.025 Na-SDS	FR = 99.20%	[113]

## Data Availability

Not applicable.
